# Comparison of Maceration and Ultrasonication for Green Extraction of Phenolic Acids from *Echinacea purpurea* Aerial Parts

**DOI:** 10.3390/molecules25215142

**Published:** 2020-11-05

**Authors:** Plamen Momchev, Petar Ciganović, Mario Jug, Eva Marguí, Jasna Jablan, Marijana Zovko Končić

**Affiliations:** 1 Faculty of Pharmacy and Biochemistry, University of Zagreb, A. Kovačića 1, 10000 Zagreb, Croatia; momchevplamen@gmail.com (P.M.); petar.ciganovic@me.com (P.C.); mjug@pharma.hr (M.J.); jjablan@pharma.hr (J.J.); 2 Faculty of Pharmacy, Medical University-Sofia, Bul. “Acad. Ivan Geshov” 15, 1000 Sofia, Bulgaria; 3 Department of Chemistry, Faculty of Sciences, University of Girona, C/M. Aurèlia Campmany 69, 17003 Girona, Spain; eva.margui@udg.edu

**Keywords:** antioxidant, *Echinacea purpurea*, glycerol, green extraction, phenolic acids

## Abstract

*Echinacea purpurea* is used in herbal medicinal products for the prevention and treatment of the common cold, as well as for skin disorders and minor wounds. In this study, the efficiency of traditional maceration using water and ethanol was compared with the maceration using mixtures of water and glycerol, a non-toxic, biodegradable solvent from renewable sources. It was found that the glycerol–water mixtures were as effective as ethanol/water mixtures for the extraction of caffeic acid derivatives. All the prepared extracts demonstrated notable antiradical properties. Furthermore, an efficient ultrasound-assisted extraction using glycerol–water mixtures was developed using six independent variables. Their levels needed for the maximum extraction of caffeic acid derivatives were as follows: glycerol 90% (*m/m*), temperature 70 °C, ultrasound power 72 W, time 40 min, and ascorbic acid 0 mg/mL. Under the optimized conditions, ultrasound-assisted extraction was superior to maceration. It achieved significantly higher yields of phenolic acids in shorter extraction time. The presence of zinc in plant material may contribute to the beneficial effects of *E. purpurea* preparations. Since glycerol is a non-toxic solvent with humectant properties, the prepared extracts can be directly used for the preparation of cosmetics or oral pharmaceutical formulations without the need for solvent removal.

## 1. Introduction

The use of plants for medicinal and cosmetic applications is undergoing an unprecedented rise. For example, over 200 official monographs with scientific and regulatory standards related to the efficacy and safety of medicinal herbal preparations in the European Union have been published so far, and the number is constantly growing. The indications for such preparations include a variety of specific applications, such as skin, sleep, gastrointestinal, and circulatory disorders [1], as well as a broad spectrum of less specific activities, such as antioxidant and anti-inflammatory activities [2]. In addition to displaying the desired activity and safety profile, modern phytopharmaceuticals and cosmetics should fulfill additional requirements, such as the appropriate stability and sensory properties. Furthermore, new concerns about the environmental impact and animal welfare are constantly emerging and new products are being developed in order to meet such needs [3].

One of the emerging research areas of cosmetic and phytopharmaceuticals research is the design of green and sustainable methods for the extraction of bioactive natural products for medicinal and cosmetic purposes. Besides the high yield of the desired natural product, the ideal extraction procedure should have low energy consumption and employ biodegradable, non-toxic, and non-flammable solvents [4,5]. One such solvent is glycerol, a natural, low-cost, non-toxic, biodegradable liquid. It is manufactured from renewable sources, e.g., as a by-product of biodiesel production [6]. An additional advantage of glycerol is its hygroscopic nature, which makes it one of the most widely used ingredients in creams and lotions, where it acts as a natural humectant, denaturant, fragrance ingredient, hair conditioning agent, oral care agent, skin protectant, and viscosity decreasing agent [7]. Furthermore, it is often used in cough syrups, as a solvent, or as a thickening agent. However, glycerol also significantly contributes to the efficacy of cough syrups due to its special properties of lubrication, demulcent effects, sweetness, and humectant activity [8]. Since the glycerol used for the extraction may easily be incorporated into the final product, glycerolic extraction of medicinal plants is very desirable from an energy saving point of view [4]. Interestingly, in spite of the numerous advantages of glycerol as an extraction solvent, it is relatively underutilized in the production of extracts for pharmaceutical and cosmeceutical purposes. Some newer literature examples of glycerol use for extraction of natural products include the extraction of saponins and polyphenols from licorice [9], alkaloids from barberry bark [10], as well as polyphenols from bran rice [11] and walnut [12].

*Echinacea purpurea* (L.) Moench (Asteraceae) (purple coneflower) is a perennial medicinal herb with important immunostimulatory and anti-inflammatory properties. According to the European Medicines Agency, *E. purpurea* and preparations thereof may be used in herbal medicinal products for prevention and treatment of the common cold, as well as for alleviation of skin disorders and minor wounds [13]. In addition, numerous scientific studies have demonstrated antioxidant, antimicrobial, antianxiety, antidepression, cytotoxicity, and antimutagenicity activities of *E. purpurea* [14,15]. Some of those activities may be related to the anti-inflammatory activity of *Echinacea* extracts, which is based on cyclooxygenase-1 (COX-1), cyclooxygenase-2 (COX-2), and 5-lipoxygenase inhibition (LOX) [16]. *E. purpurea* aerial parts contain diverse bioactive phytochemicals, including essential oils, polysaccharides, nitrogen compounds (such as alkylamides and small amounts of alkaloids), and numerous bioactive phenolics. Among these, phenolic acids are among the most prominent ones [17]. Due to the numerous health benefits that phenolic acids display, they are used to estimate the quality of raw herbal materials and their preparations according to the European Pharmacopoeia [18].

The most important phenolic acids in *E. purpurea* are derivatives of caffeic acid. Cichoric acid is the most abundant among them. It exhibits a wide array of activities, such as antidiabetic, antiviral, antioxidant, anti-inflammatory, neuroprotective, and obesity prevention activities [19]. In particular, various studies on different models have found that cichoric acid may ameliorate inflammation induced by lipopolysaccharides (LPSs) in both cell cultures and mice. Reduced inflammation was associated with downregulation of nuclear factor κB (NF-κB) and tumor necrosis factor α (TNF-α), two major regulators of inflammation responses. Several other proinflammatory factors, including nitric oxide synthase, COX-2, prostaglandin E2, interleukin-1β (IL-1β), IL-12, and IL-18, have also been reported to be downregulated by chicoric acid [19]. In addition, cichoric acid may augment the immune response through the modulation of the CD28/CTLA-4 and Th1 pathways [20].

Caftaric acid, another caffeic acid derivative present in *E. purpurea,* acts as an antioxidant, anti-inflammatory, antimutagenic, and anticarcinogenic agent [21]. Caftaric acid was shown to be a competitive tyrosinase inhibitor, making it suitable for inclusion in cosmetic products with skin whitening properties [22]. Other phenolic acid derivatives may also add to the beneficial effect on wound healing. For example, chlorogenic acid, a caffeic acid derivative, plays several important therapeutic roles, such as having antioxidant activity, as well as antibacterial, hepatoprotective, cardioprotective, anti-inflammatory, antipyretic, neuroprotective, antiobesity, antiviral, antimicrobial, antihypertensive, and central nervous system stimulating effects [23].

Bearing in mind the importance of glycerol in the pharmaceutical and cosmetic industry, as well as the beneficial effects exerted by phenolic acids present in *E. purpurea*, the aim of this work was to compare and optimize maceration and ultrasound-assisted extraction (UAE) of phenolic acids from *E. purpurea* aerial parts using glycerol, a non-toxic and ecofriendly solvent.

## 2. Results

### 2.1. Macerations

The macerations were performed using protic solvents of different polarities and viscosities, allowing for the comparison of glycerol extraction with the more common ethanol/water extraction. The conditions used for the macerations are presented in Table 1.

**Figure 1:**
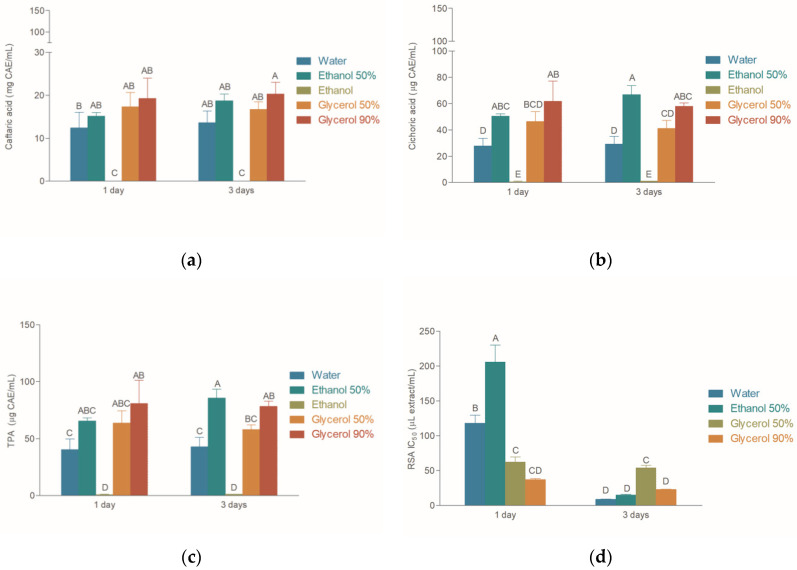
Contents of phenolic acids: (**a**) caftaric acid; (**b**) cichoric acid; (**c**) total phenolic acids (TPA); (**d**) radical scavenging activity (RSA) of the extracts prepared by maceration.

While pure water and ethanol were suitable for the extraction, it was not possible to use pure glycerol due to its high viscosity. Therefore, 90% (*m/m*) glycerol was used instead. In order to investigate the influence of time on the composition of the extracts, macerations were performed for either 1 or 3 days. The contents of phenolic acids in the extracts are shown in Figure 1.

While the extracts were rich in cichoric and caftaric acids, the amount of chlorogenic acid in the extract was below limit of detection (LOD). Thus, it was omitted from Figure 1. Generally, cichoric acid was the most abundant phenolic acid in the extracts. Its concentration ranged between 2.1 (in W-3D)- and 3.3 (in E50-1D)-fold higher than the amount of caftaric acid in the corresponding extracts. Thus, the cichoric acid content was the main contributing factor to the total phenolic acid content (TPA), calculated as the sum of caftaric, chlorogenic, and cichoric acid contents. The concentrations of caftaric and cichoric acids correlated significantly (*r*^2^ = 0.9346), implying that similar parameters affected their concentrations in the extracts.

The amounts of caftaric acids in the extracts varied greatly depending on the solvent used for the extraction. For example, E-1D and E-3D did not contain any detectable amounts of caftaric acid, while G90-1D and G90-3D contained about 20 μg/mL of phenolic acid. Similarly, in E-1D and E-3D, cichoric acid was present in very low amounts, while its content in G90-1D and E50-3D reached over 60 μg/mL. While the maceration duration did not significantly affect the contents of the target compounds, the influence of the solvent on the extraction efficiency was observable but rather low. The extraction efficiency levels for 50% ethanol, 50% glycerol, and 90% glycerol did not statistically differ from one another.

### 2.2. Radical Scavenging Activity

The radical scavenging activity (RSA) results for the extracts prepared by maceration are presented in Figure 1d. The extract prepared using ethanol did not display any observable RSA, and thus is not presented in the figure. Unlike the phenolic acid content, the radical scavenging activity of the extracts was influenced by the solvent, and in some cases the duration of the maceration. After one day of maceration, water and 50% ethanol yielded the extracts with the highest IC_50_ values (and thus the lowest RSA), while the activity levels of 50% and 90% glycerol extracts were more pronounced. However, the activity levels of the 50% ethanol and water extracts prepared by 3-day maceration were significantly better than their 1 day counterparts. On the other hand, the activity levels of 50% and 90% glycerol extracts did not significantly change with prolonged maceration. The 3 day water extract was the best radical scavenger in the study, with an RSA IC_50_ value of 8.79 μg/mL ± 0.44 μg/mL, followed by the 50% ethanol (RSA IC_50_ = 15.00 μg/mL ± 0.96 μg/mL) and 90% glycerol (RSA IC_50_ = 22.88 μg/mL ± 0.41 μg/mL) extract. The RSA activity levels of these three extracts did not statistically differ (Dunnett’s test) from the activity of positive control, butylated hydroxyanisole (BHA), with an RSA IC_50_ value of 6.12 μg/mL ± 0.17 μg/mL. In the present study, the RSA and the contents of phenolic acids were not correlated (*r*^2^ < 0.1).

### 2.3. Effects of UAE Variables on Phenolic Acid Extraction Yield

In this work, the efforts were undertaken to optimize the UAE of phenolic acids from *E. purpurea*. For this purpose, a two-level factorial design was used. The effects of the glycerol concentration (A), temperature (B), ultrasound power (C), time (D), ascorbic acid concentration (E), and the amount of solvent (F) were investigated. The independent variables and their levels are presented in Table 2.

The effects of the independent variables on the amount of target substances are presented in Table 3. The results clearly show that the extraction variables have a great impact on the success of the extraction. Depending on the extraction parameters, the concentrations of caftaric and cichoric acid changed from 6.64 to 50.26 μg/mL and from 7.49 to 155.31 μg/mL, which are approximately seven- and twenty-fold increases, respectively. The levels of independent variables needed for the maximum extraction of caffeic acid derivatives were as follows: glycerol 90% (*m/m*), temperature 70 °C, ultrasound power 72 W, time 40 min, and ascorbic acid 0 mg/mL.

Cichoric acid accounted for about two-thirds of the total phenolic acids (TPA) present in the extracts. Similar to maceration findings, the concentrations of caftaric and cichoric acids correlated significantly (*r*^2^ = 0.9169). In addition, their concentrations showed a week but significant correlation with chlorogenic acid (*r*^2^ = approximately 0.7), indicating that similar factors affected their concentrations. The concentrations of chlorogenic acid ranged from below LOD to 0.94 μg/mL, and contributed to the TPA at less than 1%.

**Figure 2:**
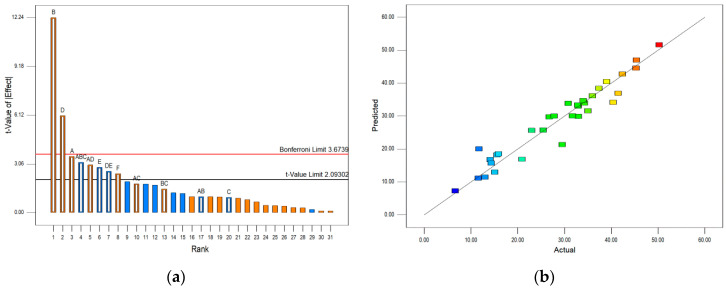
Caftaric acid content model: (**a**) Pareto chart; (**b**) actual vs. predicted results. Independent variables: A = glycerol concentration; B = temperature; C = ultrasound power; D = time; E = ascorbic acid concentration; F = amount of solvent. Blue color on the chart (**a**) indicates a negative and the orange color refers to a positive effect of independent variables. The color points on the chart (**b**) represent the value of caftaric acid (blue: lowest value; red: highest value).

**Figure 3:**
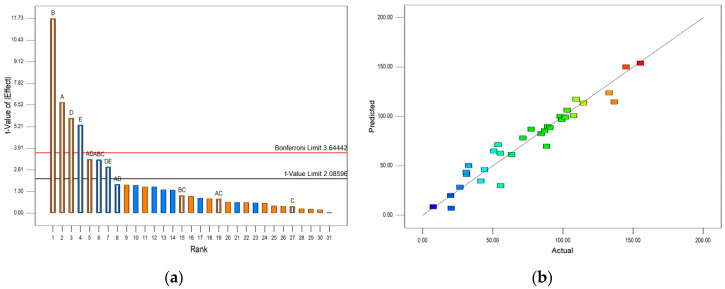
Cichoric acid content model: (**a**) Pareto chart; (**b**) actual vs. predicted results. Independent variables: A = glycerol concentration; B = temperature; C = ultrasound power; D = time; E = ascorbic acid concentration. Blue color on the chart (**a**) indicates a negative and the orange color refers to a positive effect of independent variables. The color points on the chart (**b**) represent the value of cichoric acid (blue: lowest value; red: highest value).

**Figure 4:**
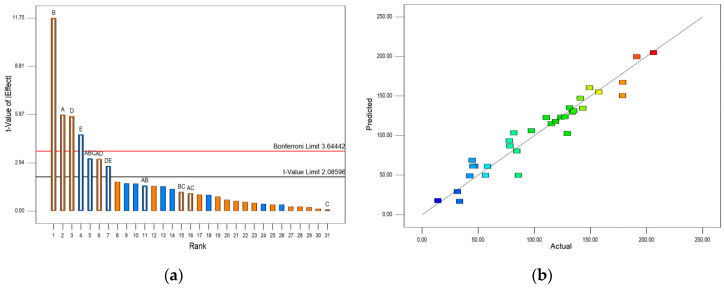
Total phenolic acid content model: (**a**) Pareto chart; (**b**) actual vs. predicted results. Independent variables: A = glycerol concentration; B = temperature; C = ultrasound power; D = time; E = ascorbic acid concentration. Blue color on the chart (**a**) indicates a negative and the orange color refers to a positive effect of independent variables. The color points on the chart (**b**) represent the value of total phenolic acid (blue: lowest value; red: highest value).

In order to characterize the significance of independent variables and to select the most significant variables based on their output responses, a Pareto chart was used. The Pareto chart depends on the standard deviation to estimate the sampling errors of variables. Two important limits in the Pareto chart are the Bonferroni limit and the *t*-value limit. Variables with coefficients above the Bonferroni limit are significant model factors. On the other hand, the terms that fall between the Bonferroni limit and the *t*-value limit are considered likely to be significant, while the coefficients below the *t*-value limit are insignificant [24]. The blue color on the charts indicates a negative and the orange color refers to a positive effect of independent variables. The ANOVA analysis confirmed that the selected models were highly significant (*P* < 0.0001), with high *r*^2^ values (>0.92), as well as confirming that only the statistically significant effects and the terms supporting the hierarchy were included in the model (details are presented in the Supplementary Materials). The Pareto charts, along with the actual vs. predicted charts for the selected responses (caftaric acid, chlorogenic acid, and TPA), are presented in Figure 2, Figure 3 and Figure 4. Due to the very low amounts of chlorogenic acid in the extracts, its chart was omitted from the analysis.

The Pareto chart of the effects of the extraction conditions on caftaric acid content (Figure 2a) shows that the factors B and D are above the Bonferroni limit (*t*-value of effect = 3.6739), and thus are significant model factors. Both of them exert positive influence on the caftaric acid content. On the other hand, not all the variables above the *t*-value limit (*t*-value of effect = 2.093) influence the content of caftaric acid positively. While its content increased together with A, F, and AD, the increase of variables E, DE, and ABC lead to the decrease of caftaric acid content. The actual vs. predicted result graph (Figure 2b) shows a good agreement between the actual values and the values predicted by the model.

The Pareto charts illustrating the effects of the extraction conditions on the contents of cichoric acid (Figure 3a) and TPA (Figure 4a) were rather similar due to the cichoric acid being the most abundant phenolic acid and contributing largely to TPA. The Bonferroni limit and the *t*-value limit were also rather similar, with the *t*-values of the effects being approximately 3.6 and 2.09, respectively. Variables with coefficients above the Bonferroni limit, such as A, B, D, and E, were significant model factors. Similarly, the terms AD, DE, and ABC, which fell between the Bonferroni limit and the *t*-value limit, were considered likely to be significant factors. The color codes indicate that A, B, D, and AD positively affected extraction efficiency, while E, DE, and ABC influenced the extraction negatively. The predicted and measured values were in good agreement (Figure 3b and Figure 4b).

### 2.4. Metal Content in E. Purpurea Aerial Parts

The contents of selected transition and second group metals were determined (Table 4). It was found that the plant material contains several elements, the presence of which may influence the anti- or pro-oxidant behavior of ascorbic acid during extraction. On the other hand, the zinc present in the sample may beneficially affect the skin- and immunity-related properties of *E. purpurea* preparations.

The plant material was especially rich in iron (255.48 mg/kg) and manganese (71.32 mg/kg). Copper, on the other hand, was present in significantly lower quantities.

## 3. Discussion

### 3.1. Phenolic Acid Contents in the Extracts Obtained by Maceration

Medicinal plants and plant extracts contain a myriad of secondary metabolites. While some of them have desirable pharmacological properties, the others may influence the overall activity of the natural extracts in either a positive or negative manner. Thus, medicinal plant extraction procedures aim to increase the amounts of desired metabolites while simultaneously decreasing the amounts of undesired or harmful ones. The amounts of secondary metabolites in the extracts depend on their physicochemical properties, extraction solvents, types of extraction, as well as on numerous extraction parameters related to the specific type of the extraction [5]. Finding the extraction procedures that yield the maximum amount of the target compound(s) with the minimum amount of the undesired ones may be a tedious, costly, and time consuming procedure.

In this work, efforts were undertaken to efficiently optimize the green extraction of bioactive phenolic acids from aerial parts of *E. purpurea* and to obtain the extracts ready to use in pharmaceutical and cosmetic products. In order to achieve this, classical maceration performed using glycerol or water mixtures was compared with maceration using ethanol, water, and mixtures thereof. Furthermore, the UAE of phenolic acids from *E. purpurea* using glycerol–water mixtures as the extraction solvent was developed.

Maceration is the oldest of the solid–liquid extraction methods and is characterized by the simplicity and low cost of the procedure, as well as by the long duration needed for the achievement of an equilibrium concentration of the extracted metabolite in the solvent [25]. The use of glycerol for maceration of phenolic secondary metabolites is relatively rare [11,26]. Bergeron et al. used glycerol for maceration of *E. purpurea* [27], but detailed and focused reports on the influence of glycerolic extraction conditions and comparisons with ethanol are still lacking.

In this work, the amount of phenolic acids extracted by using different solvents varied slightly. Although the previous research indicated that the *E. purpurea* phenolics are poorly extracted with ethanol [17], it was still interesting to note that the ethanol extracts did not contain any detectable amounts of caftaric acid, while the cichoric acid was present in very low amounts. In addition, chlorogenic acid was absent in the extracts prepared by maceration, even though Bergeron et al. [27] noted that unlike in glycerol extract, it should be present in ethanol extracts of *E. purpurea.* Previous reports indicated that 50% ethanol was the most efficient solvent for extraction of phenolic acids from potato peel (*Solanum tuberosum*) [26]. However, its extraction efficiency in this work did not statistically differ from the efficiency of the investigated glycerol–water mixtures. Bearing in mind the importance of glycerol in the cosmetic and pharmaceutical industry, as well as the aforementioned advantages of glycerol from ecological and biological points of view, this is an important finding with numerous practical implications.

### 3.2. Radical Scavenging Activity

Antioxidants in the pharmaceutical and cosmetic industry may be regarded as prophylactic and therapeutic agents. They prevent the damage caused by free radicals and other reactive oxygen species, thus hindering the pathogenesis of various disorders such as aging, cancer, diabetes, as well as cardiovascular, autoimmune, and neurodegenerative disorders [28]. Furthermore, antioxidants protect pharmaceutical and cosmetic products against the oxidation that occurs during their storage and use. Such influences include UV radiation [29], as well as free radicals- or metal-ion-induced peroxidation of polyunsaturated fatty acids, in which natural cosmetics and pharmaceuticals are especially rich [30]. In this work, the RSA of the extracts prepared by maceration was determined. The RSA of the extracts prepared by UAE was not determined, because those extracts contained ascorbic acid, a strong antioxidant, the activity of which would clearly outperform the activity of phytochemicals from *E. purpurea* and would indicate a falsely strong RSA. All the extracts prepared by maceration showed notable RSA. In general, 3 day maceration yielded the extracts with stronger RSA levels, with most 3 day extracts showing equal radical scavenging activity to BHA. The correlation between the phenolic content and the radical scavenging activity levels of the extracts was not significant. Similar observations were also reported by other authors [31]. This is not surprising, because caffeic acid derivatives are not the only substances with radical scavenging abilities in *E. purpurea*. Various other phytochemicals, which were not determined within scope of this work but are present in *E. purpurea*, such as phylloxanthobilins [32] and polysaccharides [33], may act as strong antioxidants and free radical scavengers.

### 3.3. Effect of UAE Variables on Phenolic Acid Extraction Yield

UAE is often used for extraction in solid–liquid systems because it is a simple and cost- and time-effective method, characterized by low CO_2_ emissions and solvent consumption [34]. It is especially suitable for preparation of natural extracts due to its high reproducibility and short time of extraction. The cavitation, vibration, crushing, and mixing effects in media produced by ultrasound can break the cell wall and effectively increase the mass transfer process [35,36]. An efficient UAE process should maximize the recovery of target compounds with minimal degradation, resulting in an extract with high biological activity. Ideally, this should be accomplished using “green” environmentally friendly technologies and low-cost raw materials and solvents [10]. However, in order to determine the best UAE conditions for extraction of bioactive constituents, it is often necessary to perform multiple experiments and evaluate not only the direct influence of extraction variables, but their interactions as well. In order to achieve this, a two-level factorial design with six independent variables was employed.

The selection of the solvent greatly influences the UAE extraction efficiency due to the solvent’s physical–chemical properties, such as the polarity, viscosity, and volatility. Therefore, the proportion of glycerol in water was used as the first independent variable. In accordance with their moderately polar nature, *E. purpurea* phenolic acids were best extracted using relatively high glycerol concentrations. Several studies reported that water–glycerol mixtures were more efficient extraction media than water. Examples include the UAE of chlorogenic acid and other caffeic acid derivatives from spent filter coffee [37] and polyphenols from red grape pomace [38].

In addition to the solvent, the temperature and ultrasound power may strongly affect the efficiency of UAE. High temperature and ultrasound power levels may improve the extraction process by reducing the viscosity of the solvent and by increasing the kinetic energy of the molecules in the solution. However, they may also lead to degradation of sensitive phytochemicals, including phenolic compounds [10]. Similarly, long extraction times may increase the amount of the extracted target compounds. However, long extraction times can also increase the chances of degradation of sensitive molecules. In this work, high temperature positively affected extraction. This is possibly related to the reduction of the glycerol viscosity and increase of the kinetic energy of the solvent molecules. Such an effect was observed in previous UAE glycerolic extractions of phenolics from *G. glabra* [9]. The extraction using the highest glycerol concentration seemed to be a relatively slow process. This was evidenced by the observation that the duration of the extraction exerted a positive influence on the extraction efficiency, as well as by the positive influence of the interaction of glycerol content and time. Other researchers also reported similar findings. For example, previous kinetic studies of eggplant peel extractions suggested that diffusion of phenolics in water–glycerol mixtures was slower compared with that attained with water–ethanol, but both systems had the ability to recover essentially the same levels of total polyphenols [39]. Similar results were presented in a study of the glycerolic UAE of caffeic acid derivatives from spent filter coffee [37]. It was noted in this work that the interaction of the glycerol concentration, temperature, and higher ultrasonication power exerted a negative effect on the phenolic acid extraction. This effect may be due to the generation of hydroxyl radicals, whose production is initiated by ultrasonication, especially at high temperatures [40], and their subsequent reaction with caffeic acid derivatives [41].

The influence of the amount of solvent used for the solid–liquid extraction of a fixed amount of herbal material was also assessed. In this study, the employed amounts of solvent did not significantly affect the extraction efficiency of cichoric acid or TPA. This indicates that the concentrations of the target phenolic acids did not significantly change, even when a larger volume of solvent was employed. This finding has numerous positive ecological and economical implications, because a larger amount of product may be obtained from a fixed amount of herbal material without compromising the quality of the extract. In this way, the expensive herbal material may be more efficiently utilized.

Finally, in order to impede the oxidative degradation of phenolic acids that may occur during the extraction, water-soluble antioxidant ascorbic acid was added to the reaction mixture and its influence on the phenolic acid concentration was investigated as the final independent variable. The intention of the ascorbic acid addition was to improve the extraction of phenolic acids by impeding oxidation processes that may occur during the extraction. Previous researchers have found that the addition of antioxidants to the previously prepared *E. purpurea* glycerol extracts may improve the stability of the phenolic acids present therein [27]. It is well known that ascorbic acid hinders the oxidation of chlorogenic acid [42]. Thus, it was expected that the addition of ascorbic acid to the glycerol–water extraction mixtures would have the same effect on other phenolic substances. Surprisingly, the presence of ascorbic acid in the reaction mixture had a negative influence on all of the phenolic acids analyzed in this work. The process seemed to be time-dependent, as evidenced by the negative influences of time and ascorbic acid interaction. A possible explanation is that ascorbic acid either reacted with the analyzed phenolic acids or enabled their reaction with other natural substances present in the extracts [43]. The presence of transition metal ions (e.g., ferro ions) in the solution may also be of importance, as discussed below.

In accordance with previous research [44], the results of this study showed that under the optimized conditions, UAE extraction was superior to classical maceration, because it achieved significantly higher yields of the desired phenolic acids within a much shorter extraction time than maceration. It was found that caffeic acid derivatives were best extracted using a high glycerol concentration without added ascorbic acid, a high temperature, and a low ultrasound power using a longer extraction time. This results correspond well with the described influences of independent variables. Application of those conditions led to an approximately 1.7-fold increase in caftaric acid concentration and up to a 2.6-fold increase in both the cichoric acid content and TPA in comparison with the best results achieved using the maceration protocol. Moreover, chlorogenic acid, which was absent from the extracts prepared by maceration, was present in the extracts prepared by UAE, albeit in rather low concentrations. The selected UAE variables affected the contents of targeted phenolic acids in a similar manner, which was rather expected due to their significant structural similarities. The results of this study may be used for direct preparation of the glycerol extracts suitable for use in the cosmetic and pharmaceutical industry, or for detailed investigation and optimization of the extraction using one of the designs suitable for response surface methodology, such as a Box–Behnken or central composite design.

### 3.4. Metal Contents in E. Purpurea Aerial Parts

In order to explain the observed degradation of caffeic acid derivatives in the presence of ascorbic acid, the contents of selected transition metal were assessed. Several elements, the presence of which may influence the anti- or pro-oxidant behavior of ascorbic acid during extraction, such as iron, copper, zinc, and manganese, were determined in *E. purpurea* aerial parts. It is known that ultrasonication may initiate the production of hydroxyl radicals, especially at high temperatures [40]. In addition, caffeic acid and its derivatives may react with hydroxyl radicals, forming an array of degradation products [41]. However, this effect was not pronounced enough to reduce the contents of phenolic acids in the extracts prepared without ascorbic acid. In addition to antioxidant activity, ascorbic acid in the presence of catalytic metal ions can also exert pro-oxidant effects. For example, in the Fenton reaction, ascorbic acid may enhance hydroxyl radical generation. Fe^2+^ reacts with H_2_O_2_ to generate Fe^3+^ and the hydroxyl radical. The presence of ascorbate can lead to recycling of Fe^3+^ back to Fe^2+^, which in turn will catalyze the formation of highly reactive oxidants from H_2_O_2_ [45]. Furthermore, Mn^2+^ and Zn^2+^ catalyze the reaction of ascorbic acid with oxygen by increasing the rate of radical formation, while copper promotes the oxidation and formation of free radicals of ascorbic acid, even without the presence of oxidizing agents [46]. All of these processes may generate hydroxyl and other radicals, and consequently may cause degradation of caffeic acid and its derivatives [41].

Besides possible negative effects on the UAE of phenolic acids in the presence of ascorbic acid, some metals may also display beneficial bioactive properties. Since according to European legislation, *E. purpurea* may be used in traditional medicines for prevention and treatment of the common cold and alleviation of skin disorders and minor wounds [13], the content of zinc, the metal that may support skin- and immunity-related properties of *E. purpurea*, was also determined. The micronutrient zinc is important for maintenance and development of immune cells of both the innate and adaptive immune system [47]. Furthermore, zinc deficiency has detrimental effects on wound healing [48]. While the content of zinc in the plant material was not sufficient to grant the recommended dietary allowances [49], it may likely contribute to wound healing when applied locally, as it has been found that the amount of zinc in the wound increases during the healing process. This may induce the keratinocyte proliferation, as it has been shown that keratinocyte proliferation and differentiation are controlled by zinc [50].

## 4. Materials and Methods

### 4.1. Chemicals

Reagents and standards were purchased from Sigma-Aldrich (St. Louis, MO, USA). The purity of the standards was as follows: BHA (≥98.5%), chlorogenic acid (European Pharmacopoeia Reference Standard), and gallium (99.99%). Acetonitrile was HPLC grade. Other reagents and chemicals were of analytical grade.

### 4.2. Plant Material

Plant material was supplied by the Suban company (Samobor, Croatia). The identity was confirmed by the authors using the EU pharmacopoeial monograph for *E. purpurea* [18]. A voucher specimen was deposited in the Department of Pharmacognosy, Faculty of Pharmacy and Biochemistry, University of Zagreb, Croatia (FG-2018-EPS).

### 4.3. Maceration

Powdered plant material (0.1 g) was passed through a sieve of 850 μm mesh size and suspended in 30 g of appropriate solvent, namely water, ethanol, glycerol, or their mixtures, then macerated for either 1 or 3 days in the dark (details in Table 1). Upon extraction, the mixtures were filtered and stored in the dark at −20 °C until further analysis. For each set of conditions, three independent extracts were prepared and analyzed.

### 4.4. Radical Scavenging Activity

Radical scavenging activity (RSA) was evaluated using the sTable 2,2-diphenyl-1-picrylhydrazyl (DPPH) free radical [10]. In short, DPPH solution (0.21 mg/mL, 70 μL) was added to the extract solution (130 μL). After 30 min, the absorbance was recorded at 545 nm (FLUOstar Omega, BMG Labtech, Ortenberg, Germany). DPPH solution with methanol instead of the extract served as the negative control. RSA was calculated according to the following equation:

(1) $$\mathrm{RSA}\;\left(\%\right)=\frac{A_{control}-A_{sample}}{A_{control}}\times 100$$

where *A_control_* is the absorbance of the negative control and *A_sample_* is the absorbance of the respective extract. The concentration of the extract, which scavenged 50% of the free radicals present in the solution (RSA IC_50_), was calculated. BHA was used as the standard radical scavenger. The results were expressed as μL of extract in mL of reaction solution (μL extract/mL).

### 4.5. Preparation of the Extracts According to Two-Level Factorial Design

A preliminary extraction was carried out using a two-level factorial design with the following independent variables and their ranges: glycerol concentration (10%–90%, *w/w*), temperature (20–70 °C), ultrasound power (72–720 W), time (10–40 min), ascorbic acid concentration (0–2 mg/g), and amount of solvent (10–30 g). The numbers in brackets represent the low (−1) and high (+1) limits of the corresponding variables. Detailed conditions are presented in Table 3. Powdered plant material (0.1 g) was suspended in the appropriate amount and concentration of glycerol–water mixtures with or without addition of ascorbic acid in a 50 mL Erlenmeyer flask. The extraction was performed in an ultrasonic bath (Bandelin SONOREX^®^ Digital 10 P DK 156 BP, Berlin, Germany) using the frequency of 35 Hz at various temperatures, ultrasonication strengths, and time intervals. Upon extraction, the mixtures were filtered and stored in the dark at −20 °C until further analysis.

### 4.6. RP-HPLC-DAD Determinations of Phenolic Acids

**Figure 5:**
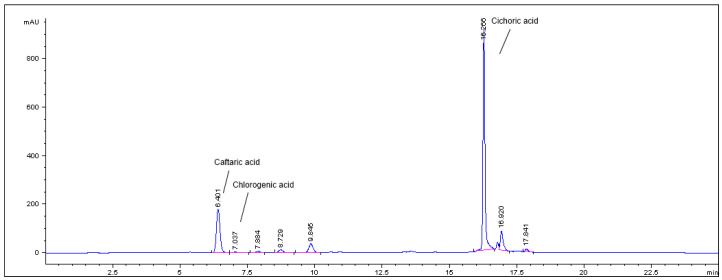
An example of a chromatogram (run 21) recorded at 330 nm.

For determination of phenolic acids, the modified European Pharmacopoeia method [18] was used. Prior to the analysis, the extracts and the standard used for the construction of the calibration curve (chlorogenic acid, 0.025 mg/mL in 70% ethanol) were filtered through a 0.45 μm PTFE syringe filter. Quantifications were performed using an HPLC instrument (Agilent 1200 series, Agilent Technologies, Santa Clara, CA, USA) equipped with an autosampler and a DAD detector. Separation was performed on a Zorbax Eclipse XDB-C18 column (5 µm, 12.5 mm × 4.6 mm, Agilent, Santa Clara, CA, USA). A mixture of phosphoric acid and water (1:999 *V*/*V*) was used as mobile phase A, while acetonitrile was used as mobile phase B. Separation was performed at 35 °C using a flow rate of 1.5 mL/min according to the following protocol: 0–13 min (90%–78% A), 13–14 min (78%–60% A), and 14–20 min (60%–40% A). Quantification was carried out at 330 nm. The calibration curve of chlorogenic acid with the corresponding coefficient of determination (*r*^2^) was y = 1834.03x + 7.12 (*r*^2^ = 0.99964), where y is the absorbance at 330 nm and x is the weight of the analyte (μg). The limit of detection (LOD) and limit of quantification (LOQ), determined according to [51], were 0.0314 μ and 0.095 μg, respectively. Retention times (t_R_) of the analytes were 6.40 ± 0.01, 7.03 ± 0.02, and 16.27 ± 0.01 min for caftaric, chlorogenic, and cichoric acids, respectively. The contents of caftaric and cichoric acids were calculated as chlorogenic acid equivalents (CAE). Total phenolic acids (TPA) were calculated as the sum of caftaric, chlorogenic, and cichoric acid contents. An example of a chromatogram is presented in Figure 5.

### 4.7. TXRF Determination of Metals in the Plant Material

TXRF analysis was performed using a commercial benchtop S2 PICOFOX TXRF spectrometer (BrukerNano, GmbH, Berlin, Germany) equipped with a low-power tungsten X-ray tube (50 kV, 1 mA) and a silicon drift detector (SDD) with a resolution < 150 eV at Mn-K_(α). The evaluation of the TXRF spectra and calculation of the analyte net peak areas were performed using Spectra Plus 5.3 software (Bruker AXS Microanalysis GmbH, Berlin, Germany) linked to the equipment. The measurement time was established as 2000 s. The vegetation samples were sieved through a sieve (diameter less of 63 μm). Sample suspensions were prepared by weighing 20 mg of sample and adding 1 mL of de-ionized water containing 10 μg of Ga as an internal standard. Duplicates were prepared for each sample and 5 min sonication in an ultrasonic bath was applied. After this, an aliquot of 10 μL of the internal standardized sample was transferred onto a quartz glass sample carrier and dried using an infrared lamp, as described in [52].

### 4.8. Statistical Analysis

The extraction experiments were planned using Design Expert software v. 8.0.6 (Stat-Ease, Minneapolis, MN, USA). The validity of the model was confirmed by the analysis of variance (ANOVA). For macerations, measurements were performed in triplicate and the results were presented as the mean ± standard deviation. Statistical comparisons were made using one-way ANOVA, followed by Tukey’s post-hoc test for multiple comparisons between extracts (JMP, SAS, San Diego, CA, USA) and Dunnett’s test for comparison with the control. *P* values < 0.05 were considered statistically significant.

## 5. Conclusions

In this work, the extraction of bioactive phenolic acids from *E. purpurea* was performed using mixtures of water with glycerol, a biodegradable, safe, affordable solvent available from renewable sources. The extracts prepared by maceration were rich in phenolic acids and potent radical scavengers. The 3 day maceration with either water, 50% ethanol, or 90% glycerol afforded extracts with activity equal to the activity of synthetic antioxidant, BHA. The UAE method, on the other hand, showed superior extraction characteristics, yielding up to 2.6-fold higher phenolic acid contents within shorter extraction times. The composition of the solvent, the time, and the temperature of the extraction significantly affected the efficiency of the extraction. Furthermore, the presence of ascorbic acid in the extraction medium lead to decreased phenolic acid contents in the prepared extracts. In addition, the presence of zinc in the plant material may contribute to the beneficial effects of *E. purpurea* preparations. Since glycerol is a non-toxic solvent with humectant properties, the prepared extracts can be directly used for preparation of cosmetics or oral pharmaceutical formulations without the need for solvent removal.

## Figures and Tables

**Table 1:** The conditions and the extracts prepared by maceration.

Extract	Solvent	Duration (Days)
W-1D	Water	1
E50-1D	Ethanol 50% (*m/m*)	1
E-1D	Ethanol	1
G50-1D	Glycerol 50% (*m/m*)	1
G90-1D	Glycerol 90% (*m/m*)	1
W-3D	Water	3
E50-3D	Ethanol 50% (*m/m*)	3
E-3D	Ethanol	3
G50-3D	Glycerol 50% (*m/m*)	3
G90-3D	Glycerol 90% (*m/m*)	3

**Table 2:** The independent variables and their levels for the two-level factorial design.

Factor Code	Factor	Units	Minimum (−1)	Maximum (+1)
A	Glycerol concentration	% (*w/w*)	10	90
B	Temperature	°C	20	70
C	Ultrasound power	W	72	720
D	Time	min	10	40
E	Ascorbic acid concentration	mg/g	0	2
F	Amount of solvent	g	10	30

**Table 3:** Independent variables, their levels for the two-level factorial design, and the responses obtained.

Std	Run	A (%, *w/w*)	B (°C)	C (W)	D (min)	E (mg/g)	F (g)	CFTA (CAE μg/mL)	CLA (μg/mL)	CCA (CAE μg/mL)	TPA (CAE μg/mL)
26	1	90	20	72	40	2	30	23.01	<LD	55.32	78.33
17	2	10	20	72	10	2	30	29.54	0.63	55.62	85.79
14	3	90	20	720	40	0	30	41.47	0.56	136.71	178.74
24	4	90	70	720	10	2	10	31.71	0.35	86.96	119.02
31	5	10	70	720	40	2	10	32.83	0.52	77.46	110.81
19	6	10	70	72	10	2	10	27.82	0.29	53.77	81.88
25	7	10	20	72	40	2	10	15.90	<LD	26.59	42.49
5	8	10	20	720	10	0	30	11.55	<LD	19.98	31.53
3	9	10	70	72	10	0	30	30.83	<LD	84.48	115.31
8	10	90	70	720	10	0	30	34.29	0.33	99.25	133.87
22	11	90	20	720	10	2	30	11.71	<LD	32.86	44.57
21	12	10	20	720	10	2	10	6.64	<LD	7.49	14.13
32	13	90	70	720	40	2	30	42.38	0.49	114.59	157.46
6	14	90	20	720	10	0	10	20.90	<LD	63.53	84.43
18	15	90	20	72	10	2	10	15.08	<LD	41.48	56.56
30	16	90	20	720	40	2	10	25.45	0.43	71.45	97.33
1	17	10	20	72	10	0	10	15.57	<LD	31.40	46.97
9	18	10	20	72	40	0	30	26.73	0.37	50.66	77.76
23	19	10	70	720	10	2	30	35.97	0.6	90.77	127.34
29	20	10	20	720	40	2	30	13.01	<LD	20.4	33.41
16	21	90	70	720	40	0	10	45.38	0.94	145.02	191.34
7	22	10	70	720	10	0	10	32.96	0.48	98.06	131.5
27	23	10	70	72	40	2	30	40.41	0.56	88.48	129.45
20	24	90	70	72	10	2	30	34.01	0.57	89.1	123.68
2	25	90	20	72	10	0	30	14.14	<LD	44.3	58.44
15	26	10	70	720	40	0	30	45.31	0.61	132.99	178.91
11	27	10	70	72	40	0	10	37.39	0.58	103.09	141.06
28	28	90	70	72	40	2	10	39.05	0.74	109.48	149.27
4	29	90	70	72	10	0	10	35.04	0.56	107.71	143.31
12	30	90	70	72	40	0	30	50.26	0.63	155.31	206.2
10	31	90	20	72	40	0	10	32.99	0.59	101.58	135.16
13	32	10	20	720	40	0	10	14.34	<LD	30.98	45.32

Independent variables: A = glycerol concentration; B = temperature; C = ultrasound power; D = time; E = ascorbic acid concentration; F = amount of solvent. Abbreviations: <LOD = below level of detection; CAE = chlorogenic acid equivalents; CCA = cichoric acid; CFTA = caftaric acid; CLA = chlorogenic acid; TPA = total phenolic acids.

**Table 4:** Contents of selected metals in *E. purpurea* aerial parts.

Element	C (mg/kg)
Mn	71.32 ± 6.65
Fe	255.48 ± 11.75
Cu	8.07 ± 4.70
Zn	37.74 ± 0.32

## Supplementary Materials

The following are available online. Tables S1–S3: Analysis of variance (ANOVA) for the caftaric, cichoric, and total phenolic acid content models, as well as post-ANOVA and prediction equations using Design Expert software.
